# Intervention to improve adverse event reporting in the emergency department: Protocol of a systematic review and meta-analysis

**DOI:** 10.1371/journal.pone.0306885

**Published:** 2024-08-22

**Authors:** Collins Atta Poku, Jonathan Bayuo, Atswei Adzo Kwashie, Adelaide Maria Ansah Ofei

**Affiliations:** 1 School of Nursing and Midwifery, University of Ghana, Accra, Legon; 2 School of Nursing and Midwifery, Kwame Nkrumah University of Science and Technology, Kumasi, Ghana; 3 Presbyterian University, Abetifi, Ghana; SR University, INDIA

## Abstract

**Background:**

Adverse event reporting is crucial for improving patient safety and identifying areas for improvement in the emergency department. Many interventions have been employed in that regard, and have been found to increase adverse event reporting rates in various settings. All published research that studied the various interventions and their effectiveness on adverse event reporting in the Emergency Department will be reviewed in this paper.

**Methods:**

CINAHL, PubMed, Medline, Cochrane Reviews Library, EMBASE, Scopus, OVID, Science Direct and Web of Science will all be searched. Studies published since January 2000 that investigated the interventions to improve adverse event reporting will be included. Two independent reviewers will execute the selection and extraction process, and we will carry out a qualitative synthesis. A meta-analysis, if possible, will be undertaken.

**Discussion:**

The present study will summarize interventions to improve adverse event reporting. It will also determine effective approaches to enhancing adverse event reporting in the emergency department. The outcome of the study will provide novel dimensions into possible interventions to improve patient safety through adverse event reporting.

**Systematic review registration:**

Protocol registration and reporting: PROSPERO CRD42023414795.

## Background

Patient safety has been identified as an important component of quality care [1]. It is a discipline in the healthcare industry concerned with preventing, reducing or eliminating risks, errors, and/or harm to patients during the delivery of healthcare; and encompasses an integrated strategy that includes developing a safety culture, improving communication, minimizing risks, utilizing technology, and standardizing systems and procedures [2].

When discussing ways to enhance patient safety, one of the most difficult parts for both patients and healthcare providers is the healthcare system’s seeming unwillingness to learn from its errors. Too often, healthcare providers and healthcare facilities fail to notify others when an error happens, or to share what they have learned when an investigation is concluded [3]. As a result, the same mistakes or errors are repeated in various settings, and patients continue to suffer injuries as a result of these preventable errors (adverse events [AEs]). Meanwhile, a major key strategy in evaluating and improving patient safety is identifying and reporting AEs in healthcare settings. AE reporting helps to improve organizational learning and quality improvement [2].

Adverse events refer to any unintended harm or injury caused to a patient during medical care, such as medication errors, misdiagnosis, falls, and infections, rather than the being due to the patient’s underlying condition. AEs can occur in any healthcare setting, including emergency care settings. Their consequences for patients are usually serious, including prolonged hospitalization, disability, or even death [4, 5]. Studies have shown that AEs occur frequently in Emergency Departments (EDs), with rates ranging from 5.5% to 27.3% of patient visits. However, many AEs remain unreported, with estimates suggesting that only 2–4% of all AEs are reported [6, 7]. Ways to prevent AEs have been proposed in the past, and the surest way is through protocols and guidelines. These guidelines can ensure that patients receive prompt and suitable care, lowering the chance of AEs [8]. Additionally, emergency care providers are encouraged to prioritize communication and teamwork. This is to ensure everyone on the team communicates effectively to give the patient the highest quality care possible. Communication about errors in the workplace is mostly through formalized adverse event reporting (AER) [9, 10].

In EDs, AER is a vital process as it helps to identify and prevent errors, improve patient safety, and ultimately save patients’ lives. This is due to the chaotic nature of these settings. Furthermore, AER can also be used to identify and correct process inefficiencies and reduce the overall cost of care [11, 12]. The challenge of AER is underreporting caused by a lack of or inadequate awareness among healthcare providers. This is caused by fear of retribution or litigation and a lack of standardized reporting procedures. To improve this phenomenon of underreporting, emergency care providers are, therefore, tasked with taking steps to prevent adverse events from occurring [13].

Among the most important approaches to curtailing AEs is a thorough review of the incident. This is to identify any contributing factors and prevent similar events in the future [14]. These steps may include changes to protocols or guidelines, additional training for staff, or changes to the physical environment of the emergency care setting. Healthcare providers have also received regular education and training on AER, including the importance of reporting, how to recognize AERs, and the steps involved in reporting [15, 16]. Additionally, most EDs have adopted standardized reporting procedures such as electronic reporting systems to ensure all AEs are reported consistently and accurately. These measures have improved non-punitive reporting systems and open communication among healthcare providers and have also helped in identifying trends and areas for improvement in patient care [17, 18].

Despite all these measures, few studies have reported on interventions to improve AER in the literature, and their effectiveness remains largely unknown. Patients have the potential to be an invaluable source of information concerning patient safety incident reporting, but historically, efforts to learn from incident reports have been concentrated on staff-led reporting systems. Some researchers contend that patients are in a unique position to influence the efficacy and safety of their care, and recent studies have shown the viability and benefits of patient reporting safety incidents in healthcare settings [19]. This review will, therefore, review studies that investigated intervention used in improving AER in EDs and their effectiveness, centering on both staff-led reporting systems and patient-led interventions.

## Method

### Protocol registration and reporting

This protocol has been registered in the Prospective Register of Systematic Review (PROSPERO) database with registration (CRD42023414795). The review will be conducted and reported according to the Preferred Reporting Items for Systematic Reviews and Meta-Analyses Protocols (PRISMA-P) guidelines [20] as detailed in Table 1. The paper that will present the results from this review will include any modifications to the review strategy.

**Table 1:** PRISMA 2020 checklist.

Section and Topic	Item #	Checklist item	Location where an item is reported
**TITLE**			
Title	1	Identify the report as a systematic review.	1–2
**ABSTRACT**			
Abstract	2	See the PRISMA 2020 for Abstracts checklist.	2
**INTRODUCTION**			
Rationale	3	Describe the rationale for the review in the context of existing knowledge.	3
Objectives	4	Provide an explicit statement of the objective(s) or question(s) the review addresses.	4
**METHODS**			
Eligibility criteria	5	Specify the inclusion and exclusion criteria for the review and how studies were grouped for the syntheses.	4–5
Information sources	6	Specify all databases, registers, websites, organisations, reference lists and other sources searched or consulted to identify studies. Specify the date when each source was last searched or consulted.	5
Search strategy	7	Present the full search strategies for all databases, registers and websites, including any filters and limits used.	5
Selection process	8	Specify the methods used to decide whether a study met the inclusion criteria of the review, including how many reviewers screened each record and each report retrieved, whether they worked independently, and if applicable, details of automation tools used in the process.	6
Data collection process	9	Specify the methods used to collect data from reports, including how many reviewers collected data from each report, whether they worked independently, any processes for obtaining or confirming data from study investigators, and if applicable, details of automation tools used in the process.	6
Data items	10a	List and define all outcomes for which data were sought. Specify whether all results that were compatible with each outcome domain in each study were sought (e.g. for all measures, time points, analyses), and if not, the methods used to decide which results to collect.	6
	10b	List and define all other variables for which data were sought (e.g. participant and intervention characteristics, funding sources). Describe any assumptions made about any missing or unclear information.	
Study risk of bias assessment	11	Specify the methods used to assess risk of bias in the included studies, including details of the tool(s) used, how many reviewers assessed each study and whether they worked independently, and if applicable, details of automation tools used in the process.	7
Effect measures	12	Specify for each outcome the effect measure(s) (e.g. risk ratio, mean difference) used in the synthesis or presentation of results.	7
Synthesis methods	13a	Describe the processes used to decide which studies were eligible for each synthesis (e.g. tabulating the study intervention characteristics and comparing against the planned groups for each synthesis (item #5)).	7–8
	13b	Describe any methods required to prepare the data for presentation or synthesis, such as handling of missing summary statistics, or data conversions.	8
	13c	Describe any methods used to tabulate or visually display results of individual studies and syntheses.	8
	13d	Describe any methods used to synthesize results and provide a rationale for the choice(s). If meta-analysis was performed, describe the model(s), method(s) to identify the presence and extent of statistical heterogeneity, and software package(s) used.	8
	13e	Describe any methods used to explore possible causes of heterogeneity among study results (e.g. subgroup analysis, meta-regression).	8
	13f	Describe any sensitivity analyses conducted to assess robustness of the synthesized results.	8
Reporting bias assessment	14	Describe any methods used to assess risk of bias due to missing results in a synthesis (arising from reporting biases).	8
Certainty assessment	15	Describe any methods used to assess certainty (or confidence) in the body of evidence for an outcome.	8
**RESULTS**			
Study selection	16a	Describe the results of the search and selection process, from the number of records identified in the search to the number of studies included in the review, ideally using a flow diagram.	
	16b	Cite studies that might appear to meet the inclusion criteria, but which were excluded, and explain why they were excluded.	
Study characteristics	17	Cite each included study and present its characteristics.	
Risk of bias in studies	18	Present assessments of risk of bias for each included study.	
Results of individual studies	19	For all outcomes, present, for each study: (a) summary statistics for each group (where appropriate) and (b) an effect estimate and its precision (e.g. confidence/credible interval), ideally using structured tables or plots.	
Results of syntheses	20a	For each synthesis, briefly summarise the characteristics and risk of bias among contributing studies.	
	20b	Present results of all statistical syntheses conducted. If meta-analysis was done, present for each the summary estimate and its precision (e.g. confidence/credible interval) and measures of statistical heterogeneity. If comparing groups, describe the direction of the effect.	
	20c	Present results of all investigations of possible causes of heterogeneity among study results.	
	20d	Present results of all sensitivity analyses conducted to assess the robustness of the synthesized results.	
Reporting biases	21	Present assessments of risk of bias due to missing results (arising from reporting biases) for each synthesis assessed.	
Certainty of evidence	22	Present assessments of certainty (or confidence) in the body of evidence for each outcome assessed.	
**DISCUSSION**			
Discussion	23a	Provide a general interpretation of the results in the context of other evidence.	9–10
	23b	Discuss any limitations of the evidence included in the review.	
	23c	Discuss any limitations of the review processes used.	
	23d	Discuss implications of the results for practice, policy, and future research.	
**OTHER INFORMATION**			
Registration and protocol	24a	Provide registration information for the review, including register name and registration number, or state that the review was not registered.	2
	24b	Indicate where the review protocol can be accessed, or state that a protocol was not prepared.	
	24c	Describe and explain any amendments to information provided at registration or in the protocol.	10
Support	25	Describe sources of financial or non-financial support for the review, and the role of the funders or sponsors in the review.	11
Competing interests	26	Declare any competing interests of review authors.	11
Availability of data, code and other materials	27	Report which of the following are publicly available and where they can be found: template data collection forms; data extracted from included studies; data used for all analyses; analytic code; any other materials used in the review.	10

### Eligibility criteria

#### Inclusion criteria

The review will be done based on the PICOS strategy. Randomised-controlled Trials (RCTs) and Single-arm trial studies that evaluate interventions or programmes aimed at enhancing AER in the EDs will be included in the review.

*Population*. Empirical, peer-reviewed studies reporting on interventions targeting the improvement of AER in adults and children treated in the EDs will be considered for inclusion irrespective of the country of origin and/or articles that should have been published in 2000. This is justified by the fact that attention to developing systems, tools or interventions to address the challenges of AEs started around the year 2000 [21].

*Interventions*. The interventions of interest will be interventions targeting the improvement of the reporting of AEs in the EDs. Articles that describe specific interventions on AER.

*Comparison*. The comparison group will be EDs without interventions for AER.

*Outcomes*. The primary outcome of interest will be the number and types of AEs reported in the EDs.

*Study design*. The review will take into account AER interventions and programme reported in RCTs and single-arm trials.

#### Exclusion criteria

Books, editorials, commentaries, newspapers, unpublished articles, and theses will be excluded from the study. If the article reports the effect of the intervention outside the ED such as in psychiatric homes, geriatric homes, outpatient departments, intensive care units, medical-surgical management units etc, it will be excluded from the review.

#### Databases and search strategy

The EMBASE Thesaurus (Emtree), Medical Subject Headings (MeSH), and text words will be used to create literature search techniques. Following a preliminary limited search of CINAHL, EMBASE, and Medline, an analysis of the text words found in the article’s text and abstract as well as its index terms will be conducted. Table 2 is the list of keywords for the search, and it will be categorised into three concepts: emergency *department* with keywords ‘emergency room’, ‘emergency care setting’, and ’medical crises unit’; *adverse events*, with terms ‘sentinel events’, ‘errors’, ‘incidents’, ‘medication errors’, ‘patient safety incidents’, ‘adverse effects’, ‘medical errors’; *interventions* with keywords ‘strategies’, ‘programme’, and ‘best practices’. Keywords in each category will be connected with the following Boolean operators: OR, AND, NOT. This will help us in designing a search strategy specific to each data source. All included databases will be searched using the chosen keywords and index phrases. Additionally, the selected reports and reference lists of identifiable reports will be manually searched for other studies. In addition to CINAHL, EMBASE and Medline, the Cochrane Reviews Library, Scopus, OVID, ScienceDirect and Web of Science will all be included in the complete search sources. To find further papers, reference lists of pertinent articles will also be reviewed. Additionally, previous systematic reviews on a related subject will be investigated to identify potential publications for inclusion.

**Table 2:** Search strategy for CINAHL.

**#1**	Search Adverse Event Reporting [Title/Abstract]
**#2**	Search Adverse effects reporting [Title/Abstract]
**#3**	Search Adverse reaction reporting [Title/Abstract]
**#3**	Search medication errors reporting [Title/Abstract]
**#4**	Search medication administration errors [Title/Abstract]
**#5**	Search sentinel events reporting [Title/Abstract]
**#6**	Search #1 OR #2 OR #3 OR #4 OR #5
**#7**	Search Emergency Department [MeSH Terms]
**#8**	Search Nursing, Emergency room [MeSH Terms]
**#9**	Search Accident and Emergency [Title/Abstract]
**#10**	Search emerg*[Title/Abstract]
**#11**	Search #7 OR #8 OR #9 OR #10
**#12**	Search (intervention OR best practice guideline OR strategies OR programme OR programs OR services) [Title/Abstract]
**#13**	Search #6 AND #11 AND #12

(“Adverse Event Reporting” OR “Adverse effects reporting” OR “medication errors reporting” OR “medication administration errors” OR “medication errors reporting” OR “Adverse Event*” OR “Adverse effect*” OR “medication administration error*” OR “medication error*” OR “patient safety incident reporting” OR “patient safety incident”) AND (“Emergency Department” OR “Nursing, Emergency room” OR “Accident and Emergency”) AND (intervention OR “best practice guideline*” OR “best practice” OR “guideline*” OR strategies OR programme OR programs OR services)

#### Study selection

All identified studies will be exported into EndNote X9 reference management software after the initial search is finished, and duplicates will be removed. Two independent reviewers (CAP and JB) will undertake title and abstract screening separately. Full-text studies that meet inclusion criteria will be retrieved and critically reviewed by two authors. Any discrepancies identified at the full-text level will be examined by the third and fourth reviewers (AMAO and AAK).

#### Data extraction and management

The data extraction form will be designed based on the characteristics of the interventions included in the study. Two independent reviewers (CAP and JB) will extract data from the papers included in the review from the Joanna Briggs Institute’s System for the Unified Management, Assessment and Review of Information (JBI SUMARI) platform. The data extracted will include specific details about the study, methods, nature of the intervention, and outcomes of interest to the review question and specific objectives. The reviewers will discuss any differences to reach a consensus, or with a third and fourth reviewers. Each included RCT and single-arm trial will have its risk of bias determined using the second iteration of the Cochrane risk of bias instrument for randomized trials (RoB 2). Using the RoB 2 tool, six domains will be evaluated: bias resulting from the randomization process, bias resulting from deviations from the intended intervention, bias arising from the lack of outcome data, bias in the measurement of the outcome, bias in the choice of the reported result, and overall bias. Each domain’s assessment of bias risk will be indicated as “low risk”, “high risk”, or “some concerns”. After that, each study’s total bias risk will be determined [22].

#### Quality assessment/study risk of bias assessment

The Grading of Recommendations, Assessment, Development, and Evaluation (GRADE) will be used to grade the overall quality of evidence [23, 24]. Four levels of evidence—“very low”, “low”, “moderate”, and “high”—will be offered along with the results to indicate how confident a person should be in the effect estimates. In the GRADE evaluation, terms like *“risk of bias”*, *“inconsistency”*, *“indirectness”*, *“impression”*, *and “publication bias”* might decrease the magnitude of an impact, while terms like *“large magnitude of effect”*, *“all residual confounding”*, *and “dose-response gradient”* can increase the level of certainty.

Again, two independent reviewers will critically appraise the RCTs and single-arm trials to evaluate the selected studies [25]. Any disagreement between the reviewers will be first discussed and if consensus is not achieved, a third independent reviewer will be consulted.

### Effect measures

The correlation coefficient (*r*) will be used to compute the effect size index [with a 95% confidence interval (CI)]. When *r* is not available, other statistics (standardized beta coefficients, t-values) will be used to calculate the effect size using the following formulas:

r=β+.05λ,

where λ = 1 when β is nonnegative and 0 when β is negative

### Ethics consideration

A review of already published literature will serve as the foundation for the current study, hence institutional review board approval is not necessary.

### Data synthesis

The Template for Intervention Description and Replication (TIDieR) checklist according to [26] will be used to present the nature of the AEs reporting interventions. Where possible, papers will be incorporated into a statistical meta-analysis using the JBI SUMARI platform. With 95% confidence intervals surrounding the summary estimate, effect sizes will be shown as a percentage (odd ratios for dichotomous data or weighted mean differences for continuous data). Using methods from Borenstein [27], the appropriate variance, or standard error, will be computed for every study by changing the correlation coefficient to the Fisher’s z scale. Cohen’s recommendations will be used to estimate the effect size correlations and interpret them as r ≤ .10—very small effect size; 0.10 > r < 0.3—small, 0.3 ≤ r < 0.5 –moderate, and r ≥ 0.5—large.

The chi-squared and I^2^ tests, which are widely used in statistics, will be used to evaluate heterogeneity. The greater the heterogeneity between the studies, the higher the I^2^ value/percentage. If I^2^ is 50%, a fixed effects model will be used; if I^2^>50%, a random effects model will be employed to estimate the heterogeneity. Only results with a p<0.05 will be considered statistically significant.

Based on the recommendations made by Tufanaru et al [28] and Munn et al [29], the meta-analysis method and model (random or fixed effects) will be chosen. Where there is enough data to examine variations in settings and AE reporting, subgroup analysis will be performed. Sensitivity studies will be carried out to evaluate choices made concerning incomplete data or limited sample size. The presented results will be used to determine whether there is high heterogeneity. Meta-analysis will not be done if the degree of heterogeneity between studies is too high. The results will be provided in narrative form, with tables and figures where needed to help with data presentation, when statistical pooling is not available.

**Fig 1:**
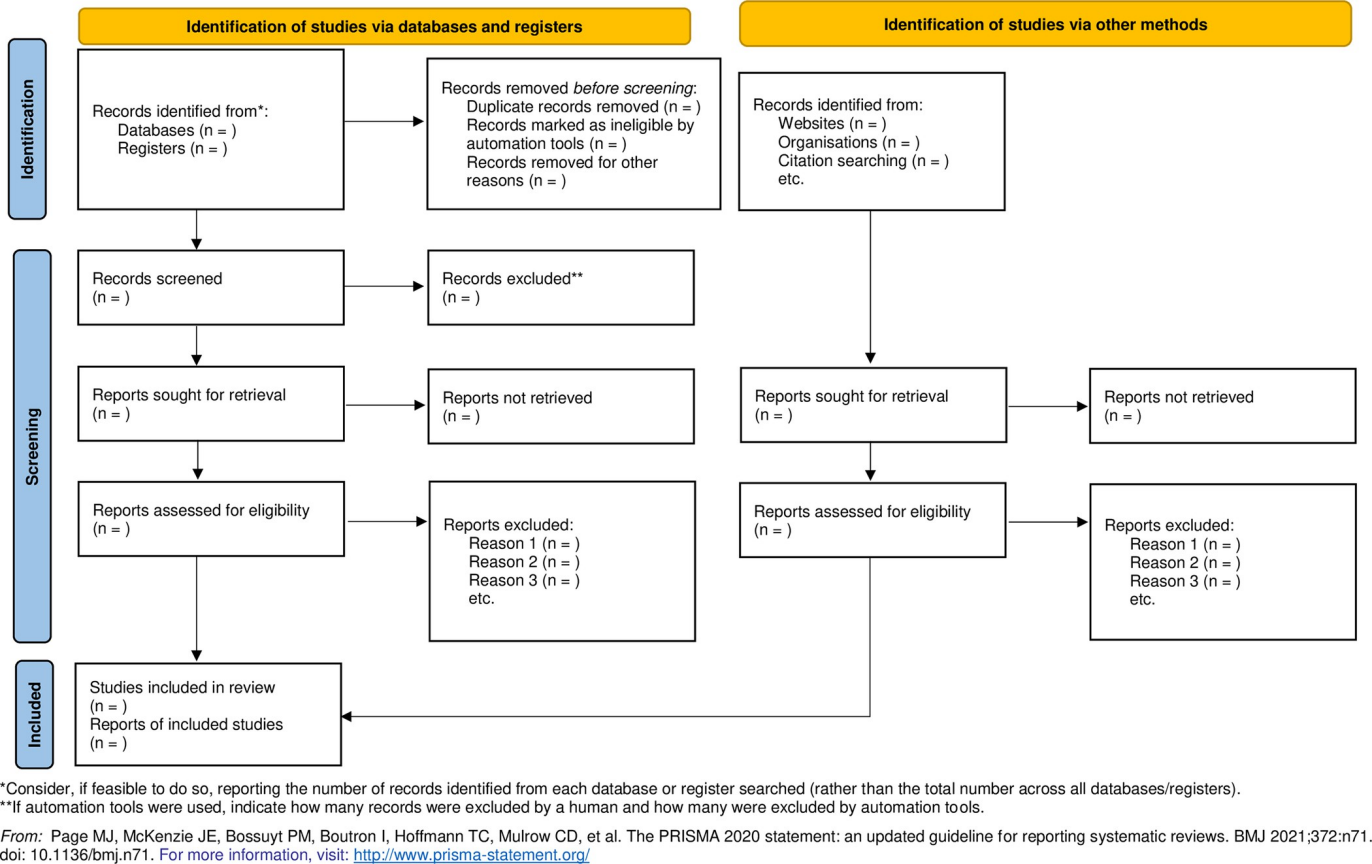
PRISMA 2020 flow diagram for new systematic reviews.

Fig 1 is the PRISMA 2020 flow diagram for new systematic reviews that will be used to present the study identification, screening and inclusion. The relationship and conclusions both within and between the selected studies will be explored in the narrative synthesis

### Reporting bias assessment

If there are ten or more studies available, funnel plots will be used to investigate the possibility of publication bias. The statistical tests for funnel plot asymmetry like the Egger test and Begg test will be run where applicable [30, 31].

## Discussion

Adverse event reporting is a critical process in healthcare systems that helps to identify and address potential patient safety issues. However, AEs are often underreported, leading to the potential for preventable harm to patients going unidentified. In the context of emergency care, interventions improve AER by addressing the barriers to reporting and encouraging healthcare professionals to report AEs.

A key intervention that has been shown to improve AER is the implementation of a patient safety culture. A patient safety culture supports the reporting of AEs and the improvement of patient safety. Hospitals with a positive patient safety culture had a higher rate of AER compared to hospitals with a negative culture [32, 33]. Specifically, hospitals with a positive culture had a median of 86.5% of AER, while hospitals with a negative culture had a median of 47.2% of AER.

Additionally, the use of electronic reporting systems has been identified as an intervention that can improve AER. Electronic reporting systems make reporting easier and more efficient for healthcare professionals to report AEs [34]. The use of an electronic reporting system increased AER by 6.2-fold compared to paper-based reporting systems. Specifically, the electronic reporting system was associated with an increase in the reporting of medication errors, falls, and pressure ulcers [35, 36].

Furthermore, education and training of healthcare professionals on the importance of AER and the reporting process can also improve reporting rates. An educational intervention aimed at increasing reporting rates of medication errors resulted in a significant increase by healthcare professionals from 10.7% to 54.9% after the educational intervention [37–39].

The ED presents their challenges of increased workload, over-crowding and chaotic nature of service delivery. More attention is paid to the survival of patients instead of patient safety of the patients [40, 41]. Even though these interventions have helped in improving AER rates and identifying potential safety issues in healthcare in general, interventions in emergency care settings are low. We expect that this study will generate interest regarding the interventions in the emergency care setting, and will contribute to the development of effective interventions to address the challenges of AE reporting to promote organizational learning.

A potential limitation that we foresee relates to the broad nature of the AE concept. The focus on interventions may also imply that qualitative data which could explain the subjective aspect of AER will be lacking. The reviewers will establish specific inclusion and exclusion criteria to narrow the focus. Thus, the reviewers will clearly define the scope and types of AEs included in the review. Another limitation will be potentially reporting only positive results published than negative or null results, potentially skewing the review’s findings. The researchers intend using statistical methods (e.g., funnel plots) to assess and adjust for publication bias. Again, the reviewers will assess the quality of each study using validated tools (e.g., Cochrane risk-of-bias tool) and perform sensitivity analyses to evaluate the study quality and risk of bias on the overall findings.

## List of abbreviations

AE: Adverse Events
AER: Adverse Event Reporting
GRADE: Grading of Recommendations, Assessment, Development, and Evaluation
EDs: Emergency Department
JBI SUMARI: Joanna Briggs Institute’s System for the Unified Management, Assessment and Review of Information
PROSPERO: The International Prospective Register of Systematic Reviews
PRISMA-P: Preferred Reporting Items for Systematic Reviews and Meta-Analyses Protocol
RCTs: Randomised Controlled Trial
RoB: Risk of Bias
SR: Systematic Review
SQUIRE: Standards for Quality Improvement Reporting
TIDieR: Template for Intervention Description and Replication
TREND: Transparent Reporting of Evaluations with Nonrandomised Designs
